# Encrypted audio dataset based on the Collatz conjecture

**DOI:** 10.1016/j.dib.2019.104537

**Published:** 2019-09-17

**Authors:** Diego Renza, Sebastian Mendoza, Dora M. Ballesteros L

**Affiliations:** Universidad Militar Nueva Granada, Colombia

**Keywords:** Audio, Encryption, Security, Cryptanalysis, Privacy, Collatz conjecture, Dataset

## Abstract

In information security, one way to keep a secret content is through encryption. The objective is to alter the content so that it is not intelligible, and therefore only the intended user can reveal the secret content. With the aim to provide examples of encrypted audio data, we applied a novel method of encryption based on the Collatz conjecture in five hundred speech recordings (50 speakers, 10 different messages), and then five hundred encrypted audio files were obtained. The main characteristics of our encrypted recordings are as follows: the spectrogram is quasi-uniform, histograms have a repetitive pattern, average of samples is around −0.4, standard deviation is around 0.55; Shannon entropy is around 7.5 (for 8-bits per sample). The novelty of the results consists in obtaining a completely different behavior than natural speech recordings, i.e.: spectrogram with higher energy in low frequencies, histogram with Gaussian behavior, average of samples around 0, standard deviation around 0.11, entropy around 5.5. A more comprehensive analysis of our encrypted signals may be obtained from the article “*High-uncertainty audio signal encryption based on the Collatz conjecture*” in the Journal of Information Security and Applications.

**Table:** Specifications Table

Subject area	*Computer science*
More specific subject area	*Speech processing; security*
Type of data	*Audio files and a spreadsheet*
How data was acquired	*The encrypted audio files were obtained through the Matlab implementation of the algorithm proposed in**Ref.* [2]
Data format	*Raw: encrypted audio files (wav)* *Analyzed data (xlsx)*
Experimental factors	*50 speakers, 10 different messages*
Experimental features	*All encrypted data were obtained with the same encryption process*
Data source location	*Colombia*
Data accessibility	*Repository name: Mendeley* *Data name: Encrypted audio files*[1] *Direct URL to data:*https://doi.org/10.17632/3vwwv3xhhc.1
Related research article	*D. Renza, S. Mendoza, D.M. Ballesteros L., High-uncertainty audio signal encryption based on the Collatz conjecture, Journal of Information Security and Applications, Volume 46, 2019, Pages 62–69*[2]

**Table:** 

**Value of the Data** - This data set can be used for cryptanalysis purposes in order to try to break the encryption method proposed in Ref. [2]. - It is useful for comparing the quality of encrypted audios in terms of their statistics like average, standard deviation, kurtosis, and entropy. Our encrypted audio files have the following values: average around −0.4, standard deviation around 0.55, kurtosis around 2.03, and entropy of 7.5 (for 8 bits). - In addition, it can be used to compare the behavior of the encrypted audio signal in terms of its spectrogram (quasi-uniform) and histogram (repetitive pattern).

## 1 Data

The shared data contain 500 audio files that have been encrypted using the algorithm proposed in Ref. [2]. The original audio recordings are 500 audio files corresponding to 50 speakers and 10 different messages per speaker. The encrypted files are new audio signals with unintelligible content that can be used to test cryptanalysis techniques; they have a length in the range [85 295] s, a sampling frequency of 8 kHz and 32 bits/sample (128 kbps).

**Fig. 1:**
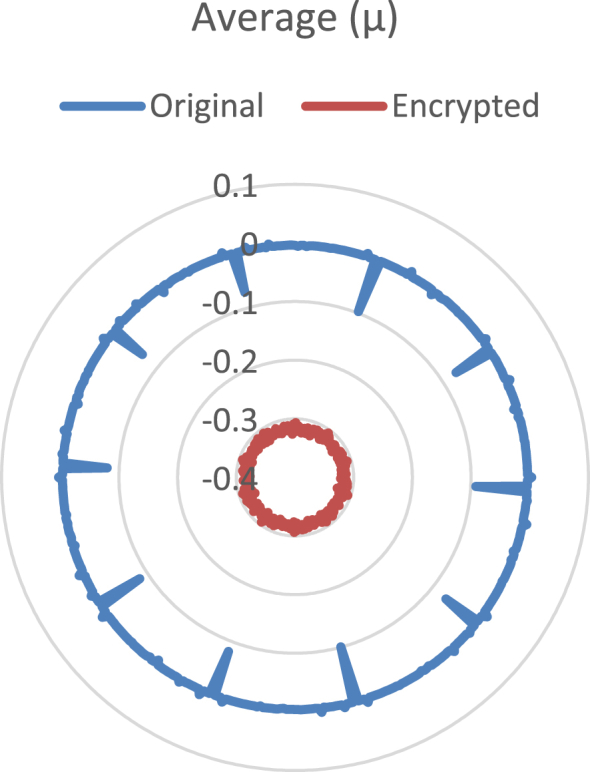
Radial plot of the mean. The blue line corresponds to the original audio files, the red line to the encrypted data.

**Fig. 2:**
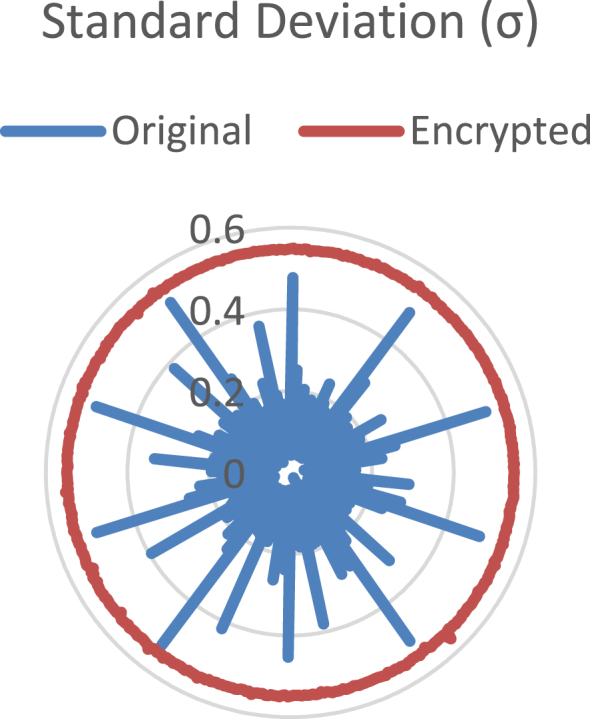
Radial plot of the standard deviation. The blue line corresponds to the original audio data, the red line to the encrypted data.

**Fig. 3:**
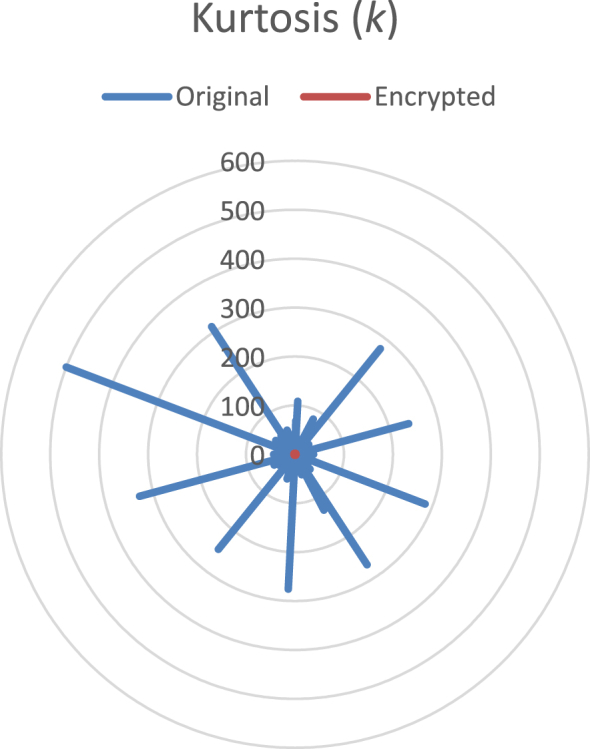
Radial plot of the kurtosis. The blue line corresponds to the original audio files; the red line to the encrypted data.

**Fig. 4:**
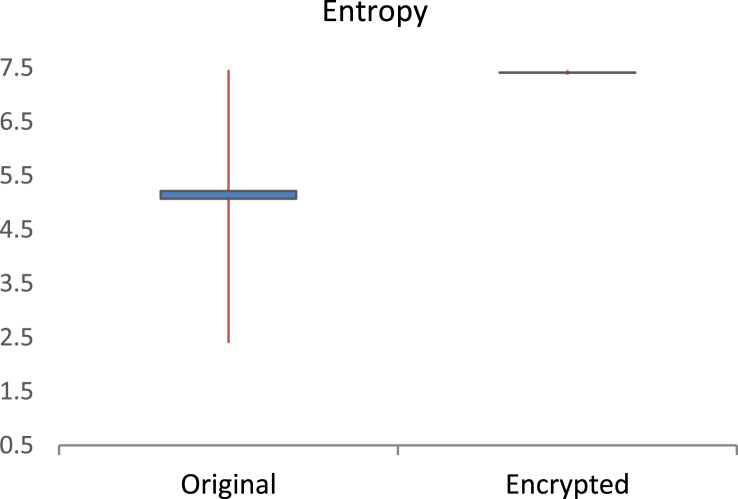
Confidence plot (95%). The left part corresponds to the entropy of the original data; the right part to the encrypted data. These values are associated to the 500 audio files of this dataset.

Statistical analysis of the audio encrypted data are provided in Fig. 1 to Fig. 4, using radial plots. Fig. 1 shows the average, Fig. 2 the standard deviation, Fig. 3 the kurtosis and Fig. 4 the entropy.

## 2 Experimental design, materials, and methods

The encrypted audio data were obtained from 500 speech recordings using the method presented in Ref. [2]. The statistical analysis of the 500 encrypted audio files is provided in the file titled *Encrypted audios.rar*.

The average, μ, is calculated with the equation $\mu =\frac{1}{n}\sum_{i=1}^{n}x_{i}$, where *x*_*i*_ are the audio samples (original or encrypted) and *n* is its number of samples. Fig. 1 shows the results of average for every group of audio file (i.e. 500 original audios, 500 encrypted audios). According to Fig. 1, the average of the original audios is around 0, but for the encrypted audio files it is around −0.4. The difference in this statistical metric between the original and its encrypted audio using the Collatz Conjecture is remarkable.

Standard deviation (σ) is obtained as $\sigma =\sqrt{\sum_{i=1}^{n}{\left|x_{i}-\mu \right|}^{2}/\left(n-1\right)}$. Fig. 2 shows the results for this parameter. It is remarkable that σ values of the original audio signals are not nearly constant, but they are for the encrypted audios.

Kurtosis is obtained through the equation, $k=E{\left(x-\mu \right)}^{4}/\sigma^{4}$, where *E(.)* represents the expected value of data. Fig. 3 shows the results of original recordings and their encrypted files. Again, the behavior between these two groups is completely different.

In terms of the Shannon entropy, H(x), is obtained as $H\left(x\right)=-\sum_{i}^{n}P\left(x_{i}\right)log_{2}\left(P\left(x_{i}\right)\right)$, where P (.) is the probability of data. For data with uniform distribution, i.e. where all values are equally likely, the expected entropy value is equal to the number of bits per sample [3]. Otherwise, entropy decreases. Fig. 4 shows the entropy comparison.

Given that, the encrypted audio files have 8 bits per sample, the theoretical highest value of entropy is 8. According to Fig. 4., the entropy of the encrypted data is around 7.5, whereas for natural audio signals it is around 5.5, in most of the cases. The entropy obtained in the encrypted audio files suggests that the level of uncertainty is very close to the highest possible.

With the encrypted recordings, the histogram and spectrogram can be obtained. If you use Matlab, the following code can help you to plot the figures:

**Figure:**
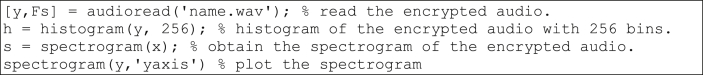

## Funding sources

This work was funded by the “Universidad Militar Nueva Granada - Vicerrectoría de Investigaciones” under the grant IMP-ING-2936.
